# Follistatin-like protein 1 plays a tumor suppressor role in clear-cell renal cell carcinoma

**DOI:** 10.1186/s40880-018-0267-2

**Published:** 2018-01-22

**Authors:** Yan Liu, Xiaojie Tan, Wenbin Liu, Xi Chen, Xiaomei Hou, Dan Shen, Yibo Ding, Jianhua Yin, Ling Wang, Hongwei Zhang, Yongwei Yu, Jianguo Hou, Timothy C. Thompson, Guangwen Cao

**Affiliations:** 1 Department of Epidemiology, Second Military Medical University, Shanghai, 200433 P. R. China; 2 Department of Urology, Changhai Hospital, Second Military Medical University, Shanghai, 200433 P. R. China; 3 Department of Pathology, Changhai Hospital, Second Military Medical University, Shanghai, 200433 P. R. China; 4 Genitourinary Medical Oncology-Research, University of Texas MD Anderson Cancer Center, Houston, TX 77030 USA

**Keywords:** Follistatin-like protein 1, Clear cell renal cell carcinoma, NF-κB, HIF-2α, Prognosis, Tumor suppressor

## Abstract

**Background:**

We previously showed that the expression of follistatin-like protein 1 (FSTL1) was significantly down-regulated in metastatic clear-cell renal cell carcinoma (ccRCC). In this study, we aimed to characterize the role of FSTL1 in the development of ccRCC.

**Methods:**

The effects of FSTL1 on cell activity and cell cycle were investigated in ccRCC cell lines with altered FSTL1 expression. Gene expression microarray assays were performed to identify the major signaling pathways affected by *FSTL1* knockdown. The expression of FSTL1 in ccRCC and its effect on postoperative prognosis were estimated in a cohort with 89 patients.

**Results:**

*FSTL1* knockdown promoted anchorage-independent growth, migration, invasion, and cell cycle of ccRCC cell lines, whereas *FSTL1* overexpression attenuated cell migration. *FSTL1* knockdown up-regulated nuclear factor-κB (NF-κB) and hypoxia-inducible factor (HIF) signaling pathways, increased epithelial-to-mesenchymal transition, up-regulated interleukin-6 expression, and promoted tumor necrosis factor-α-induced degradation of NF-κB inhibitor (IκBα) in ccRCC cell lines. FSTL1 immunostaining was selectively positive in epithelial cytoplasm in the loop of Henle, and positive rate of FSTL1 was significantly lower in ccRCC tissues than in adjacent renal tissues (*P* < 0.001). The multivariate Cox regression analysis showed that the intratumoral FSTL1 expression conferred a favorable independent prognosis with a hazard ratio of 0.325 (95% confidence interval 0.118–0.894). HIF-2α expression was negatively correlated with FSTL1 expression in ccRCC specimens (*r* = − 0.229, *P* = 0.044). Intratumoral expression of HIF-2α, rather than HIF-1α, significantly predicted an unfavorable prognosis in ccRCC (log-rank, *P* = 0.038).

**Conclusions:**

FSTL1 plays a tumor suppression role possibly via repressing the NF-κB and HIF-2α signaling pathways. To increase FSTL1 expression might be a candidate therapeutic strategy for metastatic ccRCC.

## Background

Renal cell carcinoma (RCC) is the seventh most common cancer worldwide and the tenth most common cancer in the urban areas of China [1]. Clear cell RCC (ccRCC) is the major histotype, accounting for 80% of all RCC [2]. Early-stage RCC has an asymptomatic clinical course; 25%–30% of patients appear with metastatic disease [2]. Approximately 30% of RCC patients who undergo curable resection of localized tumors eventually develop distant metastases [2]. Metastatic RCC is resistant to routine chemotherapy and radiotherapy. The 5-year survival rate of RCC patients with metastasis was 31.6% [3]. However, a percentage of metastatic RCC is sensitive to immunotherapy and targeted therapy [4–7]. To improve postoperative survival of RCC patients, it is important to identify the factors that have prognostic or predictive values, thus leading to monitoring for disease recurrence and opportunities for targeted and/or immunotherapy. D9S168 microsatellite alteration in tumors [8], intratumoral neutrophil [9], intratumoral expression of proteins including chemokine (C-X-C motif) receptor 8 (CXCR8) [10], CD44 [11], CXCR3, insulin-like growth factor mRNA-binding protein 3, survivin, B7 homolog 1 [12], glycolytic enzymes [13], circulating molecules such as human telomerase reverse transcriptase [14], spermine/spermidine [15], urinary cathepsin D [16], and co-expression of interleukin-6 (IL-6) and its receptor have been associated with poor prognosis in RCC patients [17]. High level of chemokine (C-X-C motif) ligand 16 (CXCL16) expression in tumors is associated with a longer survival [18]. However, more prognostic and/or predictive molecules are needed to optimize postoperative care for RCC patients with different genetic background.

In our previous study investigating global profiling of gene expression in RCC cells with different metastatic potential, we found that follistatin-like protein 1 (FSTL1) was frequently down-regulated in metastatic ccRCC [19]; and the C allele of rs1259293 in the coding region of *FSLT1* was associated with an increased risk and unfavorable postoperative prognosis of RCC, possibly by down-regulating *FSTL1* expression in renal tissues [20]. FSTL1, a secreted glycoprotein encoded on chromosome 3 in humans, is widely expressed in cells of non-hematopoietic lineage, particularly in cells of the mesenchymal lineage [21]. FSTL1 is induced in response to inflammatory injuries and plays important roles in promoting the accumulation of myofibroblasts and subsequent fibrosis, promoting cardiac function, and reducing glomerular and tubulointerstitial inflammatory damage in the kidney via attenuating tumor necrosis factor alpha (TNFα)-stimulated expression of proinflammatory cytokines [22–24]. The role of FSTL1 in cancers is controversial. During cancer metastasis from the primary site to the bone, FSTL1 mediates cancer cell invasion and expands a population of bone marrow-derived pluripotent mesenchymal stem-like cells [25]. In prostate cancer, the androgen-dependent up-regulation of FSTL1 promotes growth of cancer cells [26]. In colorectal cancer (CRC), FSTL1 is selectively expressed in cancer stroma and attenuates CRC cell proliferation [27]. Although FSTL1 is overexpressed in plasma and cancerous tissues of CRC patients, it has not been, thus far, implicated in prognosis [28]. In ovarian and endometrial cancers, FSTL1 functions as a tumor suppressor via inducing apoptosis [29]. However, the role of FSTL1 in RCC remains elusive.

We hypothesized that FISTL1 might play a role as tumor suppressor in ccRCC. In the present study, we aimed to clarify the effects of aberrant FSTL1 expression on the growth and aggressiveness of RCC cells, identify the signaling pathways that were affected by FSTL1, and validate the prognostic functions of FSTL1 with a cohort of RCC patients.

## Methods

### Cell culture, plasmid constructs, and transfection

Human ccRCC cell lines ACHN and 786-O were purchased from American Tissue Culture Collection (Manassas, VA, USA), with Accession Numbers CRL-1611 and CRL-1932, respectively. Human embryonic kidney (HEK) 293T cells were purchased from the cell bank, Chinese Academy of Sciences (No. CBP60439, Shanghai, China). NRCC (low-metastatic) and MRCC (high-metastatic) ccRCC cell lines were established from two Chinese ccRCC patients in our laboratory [30]. 786-O cells were grown in RPMI-1640 media (Hyclone, Pittsburgh, PA, USA) supplied with 10% fetal bovine serum (FBS) (GIBCO, Grand Island, NY, USA), 100 U/mL penicillin, and 100 μg/mL streptomycin (Invitrogen, Carlsbad, CA, USA). ACHN, MRCC, NRCC, and HEK 293T cells were grown in DMEM (Hyclone) supplied with 10% FBS, 100 U/mL penicillin, and 100 μg/mL streptomycin.

Two short hairpin RNA (shRNA) targeting the different regions of *FSTL1* mRNA (shFSTL1-1 and shFSTL1-2) and a scrambled control (shScramble) were constructed into the pSuper-retro vector (OligoEngine, Seattle, WA, USA) and confirmed by sequencing, respectively. The sequences of the shRNA were 5′-AAGAGAGTGAGCACCAAAGAG-3′ (shFSTL1-1), 5′-AAGCATCAGGAAACAGCTGAA-3′ (shFSTL1-2), and 5′-CTGGCATCGGTGTGGATGA-3′ (shScramble). A full-length human *FSTL1* cDNA clone (No. MHS4771-99611059) was purchased from Thermo Fisher Scientific (Pittsburgh, PA, USA), released by *Bam*HI and *Xho*I digestion, and inserted into the mammalian expression vector pcDNA3.1/V5-His topo (Invitrogen) to construct a topo-FSTL1 plasmid that could express FSTL1.

The retrovirus packing vector Pegpam 3e and recombination directionality factor (RDF) vector were kindly provided by Yang [31]. Retroviral supernatants were harvested at 72 h after HEK 293T cells were co-transfected with shRNA plasmids and two packing plasmids by Fugene HD Transfection Reagent (Promega, Madison, WI, USA). NRCC cells were incubated with virus-containing medium supplemented with 4 µg/mL polybrene (Sigma-Aldrich, St. Louis, MO, USA). Stable *FSTL1*-knockdown NRCC cells were selected in the presence of 3.5 µg/mL puromycin (Sigma-Aldrich) for 7 days. Topo-FSTL1 expression plasmid, empty topo vector, shFSTL1-1, shFSTL1-2, and shScramble were transfected using lipofectamine LTX and Plus Reagent (Invitrogen) into 786-O cells and ACHN cells, respectively. *FSTL1* mRNA expression was examined by quantitative reverse transcription-polymerase chain reaction (qRT-PCR) and Western blotting.

### Growth, migration, and invasion assays

Anchorage-independent growth of RCC cells with aberrant FSTL1 expression was evaluated with a double-layered soft agarose culture system, as previously described [30]. Cell migration assay (without matrigel) and cell invasion assay (with matrigel) were performed using 8-μm pore size 24-well cell culture transwell plates (Corning, Corning, NY, USA). These experiments were performed in triplicate.

### Cytometry

Cell cycle and cell surface markers of NRCC-shScramble and NRCC-shFSTL1 cells were examined using a flow cytometer (MACSQuant, Miltenyi Biotec, Bergisch Gladbach, Germany). The estimation of cell cycle was performed with propidium iodide (PI) staining as previously described [29]. To compare proportions of cells in different cell cycle phases, NRCC-shFSTL1 and NRCC-shScrambled cells were passaged synchronously. Cell markers were detected using anti-CD44-PE (1:10 dilution; Biolegend No. 338808, San Diego, CA, USA), anti-CD105-FITC (1:10 dilution; Biolegend No. 323204), anti-CD24-FITC (1:10 dilution; Biolegend No. 311104), anti-CD99-FITC (1:10 dilution; Biolegend No. 318006), anti-CD133-PE (1:10 dilution; Miltenyi No. 00029, Bergisch Gladbach, Germany), anti-vimentin (1:50 dilution; Santa Gruz Biotechnology No. Sc-32322, Santa Gruz, CA, USA), and anti-EpCAM (1:50 dilution; Cell Signaling Technology No. 2929, Danvers, MA, USA) monoclonal antibodies. Cell debris and fixation artifacts were excluded by appropriate gating. The acquisition process was stopped when 10,000 events for cell cycle analysis and 30,000 events for cell surface marker analysis were collected in the population gate. The FlowJo version 7.6 software (Flowjo, Ashland, NC, USA) was used for data acquisition and analysis. Assays for other details were as previously described [30]. Each flow cytometry assay was conducted in triplicate.

### cDNA microarray analysis

Total RNAs from NRCC-shScramble, NRCC-shFSTL1-1, and NRCC-shFSTL1-2 cells were extracted using Trizol Reagent (Life technologies, Carlsbad, CA, USA) and then reversely transcribed, biotin-labeled, fragmented, and hybridized to Human Genome U133 plus 2.0 chips (Affymetrix, Santa Clara, CA, USA) according to the manufacturer’s instructions. All hybridized microarrays were scanned by Affymetrix GeneChip^®^ Scanner 3000 (Affymetrix). Raw intensities were extracted by Command Console Software 3.1 (Affymetrix). Preprocessing was then performed by MAS5 integrated in Expression Console (Affymetrix). Probe sets with low intensities and more than two absent calls were filtered. Probe set-level data was transformed into log2 scale and summarized to gene-level data by averaging the intensities of probe sets annotating to the same gene. Genes containing probe sets whose fold changes were more than two and without absent calls were considered differentially expressed genes. Gene set enrichment analysis (GSEA) was applied to explore the enriched gene sets [32]. The gene expression differences between NRCC-shFSTL1 and NRCC-shScramble cells were computed as the statistic to rank the genes in the data. Normalized enrichment score (NES) were computed for every gene set in chemical and genetic perturbation part of Molecular Signatures Database v4.0 (MsigDB v4.0, http://software.broadinstitute.org/gsea). Gene sets were randomized 1000 times to obtain false discovery rate (FDR). Gene sets with FDR of < 0.25 were considered statistically significant. Microarray data were deposited in GEO with Accession No. GSE76948. All microarray data were analyzed using R programming (https://www.cran.r-project.org).

### qRT-PCR and Western blotting

Total RNAs were isolated and reversely transcribed into cDNA, and subjected for qRT-PCR as previously described [9]. The primers for amplification of *FSTL1*, *IL*-*6*, *E*-*cadherin*, *N*-*cadherin*, and glyceraldehyde-3-phosphate dehydrogenase (*GAPDH*) as well as their amplified conditions and locations are summarized in Table 1. Amplification efficiencies of all primers are present in Table 1, estimated based on standard curve assay as previously described [33]. Gene expression was relatively quantified using *GAPDH* as an internal control. qPT-PCR assays were controlled using no template controls (NTC) and “no-reverse transcription” (no-RT) controls. Quantification cycle (C_q_) was no less than 35 and no peak of melt curve occurred in both controls. All qPCR reactions, including reactions of NTCs and “no-RT” controls, were run in triplicate and the average C_q_ of each sample was calculated based on all three biological replicates. Each qRT-PCR assay was conducted in triplicate. Protein was extracted, quantified, and subjected to Western blotting according to standard protocols as previously described [34]. The primary antibodies used in this study were as follows: anti-FSTL1 (C-term) (1:1000 dilution; Abgent No. AP10534b, San Diego, CA, USA), anti-IκBα (1:1000 dilution; Cell Signaling Technology No. 9242), and anti-β-actin (1:1000 dilution; Cell Signaling Technology No. 3700). β-actin was used as the loading control for cellular proteins. The protein bands were detected using GBOX Chemi XL1.4 machine (Gene, Cambridge, UK) and GeneSys version 1.2.2.0 software. The densities of protein bands were quantified using Genetools software (version 4.02, Cambridge, UK).

**Table 1:** Primers and reaction conditions for the measurement of gene transcriptional levels using quantitative reverse transcription-polymerase chain reaction

Gene	Reaction program	Forward primer (5′–3′)	Reverse primer (5′–3′)	*E* (%)	*R* ^*2*^	Location
*FSTL1*	95 °C for 3 min; 45 cycles of 95 °C for 10 s, 60 °C for 10 s, and 72 °C for 25 s	AAATGCAGCTCCCTGTCCAA	ACTCTTGCCCTCCTCCCATAG	95.5	0.999	Transcript: NM_007085.4 Forward primer: exon 11 Reverse primer: exon 11
*IL*-*6*	95 °C for 10 min; 45 cycles of 95 °C for 10 s, 60 °C for 10 s, and 72 °C for 25 s	GCTTTAAGGAGTTCCTGC	GGTAAGCCTACACTTTCCA	102.6	0.997	Transcript: NM_000600.4 Forward primer: exon 5 Reverse primer: exon 5
*N*-*cadherin*	95 °C for 3 min; 45 cycles of 95 °C for 10 s, 60 °C for 10 s, and 72 °C for 25 s	TGGATGAAGATGGCATGG	AGGTGGCCACTGTGCTTAC	98.6	0.999	Transcript: NM_001792.4 Forward primer: exon 3 Reverse primer: exon 4
*E*-*cadherin*	95 °C for 4 min; 40 cycles of 95 °C for 10 s and 60 °C for 45 s	GTCATCCAACGGGAATGCA	TGATCGGTTACCGTGATCAAAA	97.1	0.999	Transcript: NM_004360.4 Forward primer: exon 4 Reverse primer: exon 5
*GAPDH*	95 °C for 10 min; 45 cycles of 95 °C for 10 s, 60 °C for 10 s, and 72 °C for 25 s	TGACTTCAACAGCGACACCCA	CACCCTGTTGCTGTAGCCAAA	101.5	0.998	Transcript: NM_002046.5 Forward primer: exon 6 Reverse primer: exon 4

E = primer efficiency; R^2^ = correlation coefficient

*FSTL1* follistatin-like protein 1; *IL-6* interleukin-6; *GAPDH* glyceraldehyde-3-phosphate dehydrogenase

### Study patients and follow-up

Formalin-fixed paraffin-embedded specimens of surgically removed tissues were collected from Changhai Hospital Affiliated to Second Military Medical University (Shanghai, China) between December 1998 and November 2011. All specimens were pathologically confirmed as ccRCC at the enrollment. We excluded ccRCC patients who refused to be enrolled in the study and those who did not adhere to the follow-up examination. All patients enrolled in this study were re-examined at our hospital within 1 month after surgery. The follow-up examination was performed every 6 months at our outpatient clinics or through phone. The last date of follow-up was May 5, 2015. Death from ccRCC relapse was defined as an event. Patients alive at the last follow-up and died of other causes were censored. Disease-specific survival (DSS) was defined as the duration from the date of receiving surgery to the date when patient died of ccRCC or when patient received the last follow-up. This study was approved by the institutional review board of Second Military Medical University. All experiments were performed in accordance with relevant guidelines. Informed consent was obtained from all subjects.

### Immunohistochemistry

Immunohistochemistry (IHC) for tumor and adjacent tissues of ccRCC patients was processed using standard techniques in our laboratory [34]. The expression levels of FSTL1 was examined in all available tumor and adjacent tissue samples. Meanwhile, hypoxia-inducible factor (HIF)-1α and HIF-2α were also examined in the patients with sufficient tissue samples. Rabbit polyclonal antibodies to human FSTL1 (C-term) (1:50 dilution; Abgent No. AP10534b), anti-HIF-1α (1:30 dilution; Novus Biologicals No. NB100-105 Littleton, CO, USA), and anti-HIF-2α antibodies (1:300 dilution; Novus Biologicals No. NB100-132) were applied according to the manufacturers’ protocols. The IHC staining was analyzed by Leica microsystems (DMI3000B, Wetzlar, Germany) and LAS version 4.0.0 software. IHC scores were independently assessed by three investigators who were blind to the clinical data. Briefly, IHC score was ranked by negative (−), slightly positive (+), moderately positive (++), and strongly positive (+++) according to the extent and intensity of immunostaining. The staining extent was graded as 1 (0%–4%), 2 (5%–24%), 3 (50%–74%), and 4 (> 75%); the staining intensity was ranked by 0 (negative), 1 (weak), 2 (moderate), and 3 (strong). Values of staining intensity and extent were multiplied as the IHC score: − (0), + (1–3), ++ (4–8), +++ (≥ 9). We accessed each pathological site of the adjacent normal specimens including glomeruli, proximal convoluted tubules, distal convoluted tubules, and collecting ducts, independently, and then summed these scores as the total score of an adjacent tissue. There was an agreement on immunoreactive scores (87%) among the investigators. Consensus was obtained after discussion.

### Statistical analysis

The comparative threshold cycle (Ct) method was employed to quantify the relative change in expression of target genes. Student *t* test was performed for two-group comparisons, and one-way ANOVA was performed for three-group comparisons. Paired Wilcoxon test was applied to evaluate the degree of positive immunostaining for FSTL1. Non-parametric analysis of Spearman correlation test was used to assess the correlation of the expression level of FSTL1 with that of HIF-1α or HIF-2α. For postoperative survival analysis, DSSs and their 95% confidence intervals (95% CIs) were estimated by Kaplan–Meier method. The log-rank test was used to compare DSSs between groups. Proportional hazard assumption was assessed by drawing Kaplan–Meier survival curves. Multivariate Cox proportional hazards model was applied to estimate the hazard ratios (HRs) with 95% CIs for DSSs. Age, gender, and FSTL1 staining, and American Joint Committee on Cancer (AJCC) stage were introduced into this model. Above statistical tests were two-sided and conducted using Statistical Program for Social Sciences (SPSS 20.0, Chicago, IL, USA). Data are presented as mean ± standard error of mean (SEM) for triplicates. A *P* value of < 0.05 was considered statistically significant.

## Results

### The growth and aggressiveness of ccRCC cells with altered expression of FSTL1

**Fig. 1:**
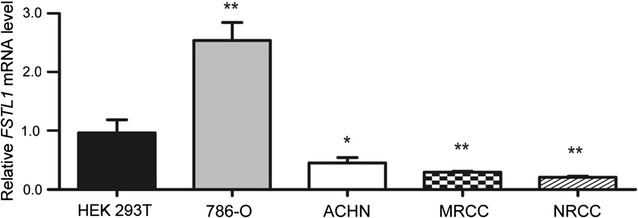
Follistatin-like protein 1 (*FSTL1*) mRNA expression levels in clear cell renal-cell carcinoma (ccRCC) and kidney cell lines. Relative levels of *FSTL1* mRNA expression in ccRCC cell lines (ACHN, NRCC, MRCC, and 786-O) and human embryonic kidney (HEK) 293T cells (*P < 0.05, **P < 0.01)

qRT-PCR was applied to determine the gene expression level of *FSTL1* in ccRCC cell lines (ACHN, NRCC, MRCC, and 786-O) and HEK 293T cells (Fig. 1). It was shown that *FSTL1* mRNA level was significantly lower in ACHN, NRCC, and MRCC cells than in HEK 293T cells (*P* values were 0.022, 0.004, and 0.007, respectively); however, *FSTL1* mRNA level was significantly higher in 786-O cells than in HEK 293T cells (*P* = 0.002).

**Fig. 2:**
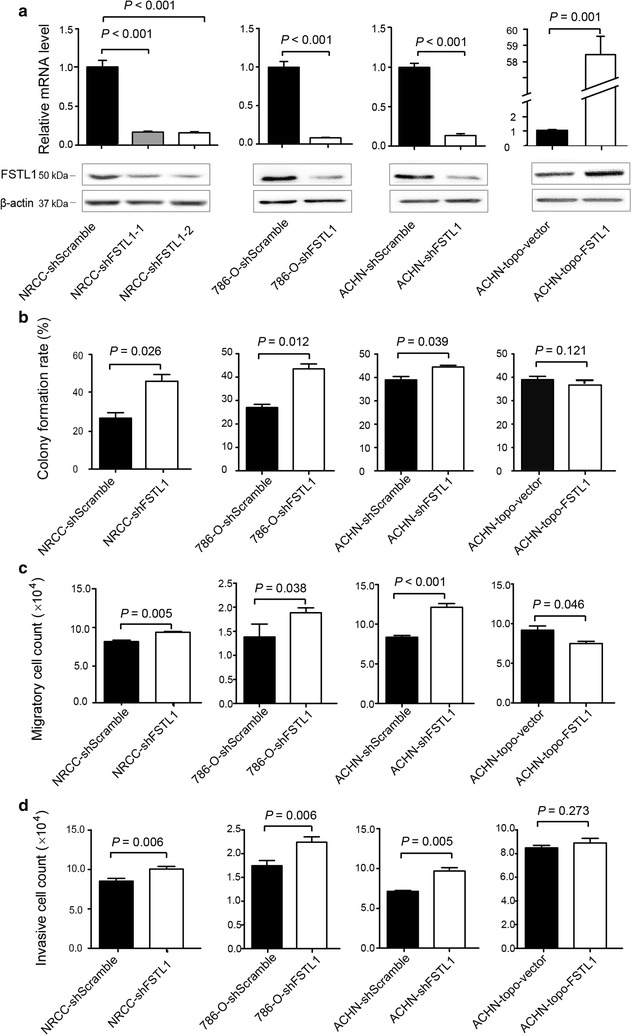
Effects of altered FSTL1 expression on anchorage-independent growth, migration, and invasion of ccRCC cells. **a** Relative level of *FSTL1* mRNA in the stable FSTL1 knockdown cell lines (NRCC-shFSTL1-1 and NRCC-shFSLT1-2), in cell lines with transient transfection of shFSTL1 (786-O-shFSTL1 and ACHN-shFSTL1), and in ACHN cell line transiently transfected with *FSTL1* cDNA (ACHN-topo-FSTL1). **b** Anchorage-independent growth of ccRCC cell lines with *FSTL1* knockdown and of ACHN cells transiently transfected with topo-FSTL1. **c** Migration of ccRCC cell lines with *FSTL1* knockdown and of ACHN cells transiently transfected with topo-FSTL1. **d** Invasion of ccRCC cell lines with *FSTL1* knockdown and of ACHN cells transiently transfected with topo-FSTL1

Retrovirus-mediated shRNA constructs targeting different regions of *FSTL1* mRNA was applied to generate two stable *FSTL1*-knockdown cell lines termed NRCC-shFSTL1-1 and NRCC-shFSTL1-2 that were derived from NRCC cells. *FSTL1* mRNA level was down-regulated by approximately 80% in NRCC-shFSTL1-1 and NRCC-shFSTL1-2 cells compared with that in shScramble control NRCC cells (Fig. 2a). Altered expression of FSTL1 was confirmed on protein levels by Western blotting (Fig. 2a). We selected NRCC-shFSTL1-2 cells in subsequent cell activity and cell cycle percentage assays. *FSTL1* knockdown significantly promoted anchorage-independent growth (Fig. 2b), migration (Fig. 2c), and invasion (Fig. 2d). Transient transfection of shFSTL1 significantly reduced *FSTL1* transcription in 786-O and ACHN cells, resulting in significantly increased anchorage-independent growth, migration, and invasion (Fig. 2). Transient transfection of *FSTL1* cDNA significantly increased *FSTL1* expression in ACHN cells and only attenuated cell migration ability (Fig. 2). MRCC was resistant to the stable or transient transfection of shFSTL1.

**Fig. 3:**
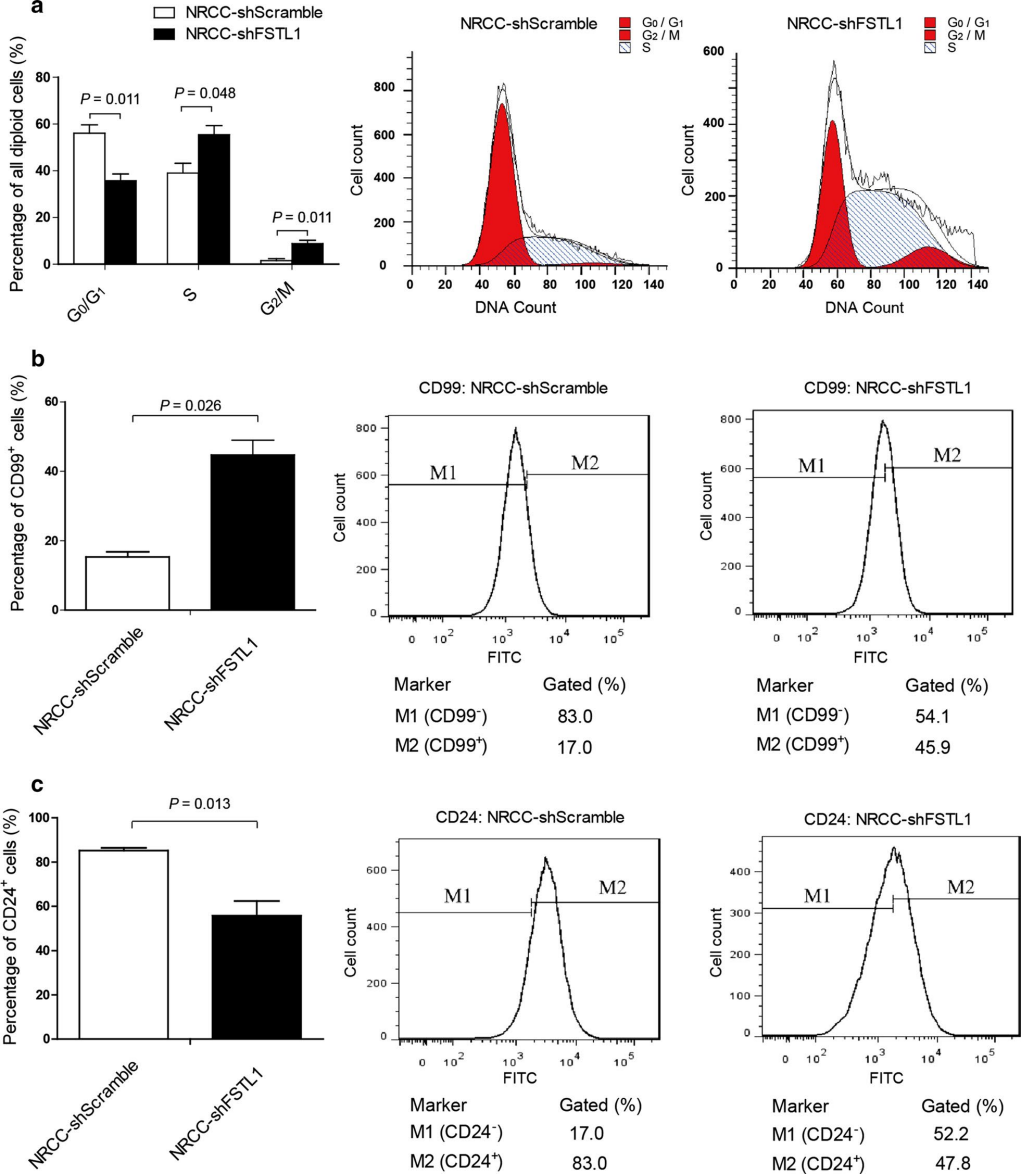
Effect of *FSTL1* knockdown on cell cycle and adhesion molecule expression in ccRCC cells. **a** Effect of *FSTL1* knockdown on cell cycle progression of NRCC cells. The percentage of cells in S and G_2_/M phases is higher in NRCC-shFSTL1 cells (55.4% ± 4.0% and 8.8% ± 1.4%) than in NRCC-shScramble cells (39.0% ± 4.2% and 1.5% ± 0.8%). The first and second peaks of the red curve represent the cell count of G_0_/G_1_ and G_2_ phases, respectively. The shaded plot reflects the cell count of S phase. **b**, **c** Effect of *FSTL1* knockdown on the expression of CD99 and CD24 on NRCC cells. The percentage of CD99-positive cells is higher in NRCC-shFSTL1 cells (44.7% ± 4.3%) than in NRCC-shScramble cells (15.3% ± 1.5%), whereas the percentage of CD24-positive cells is lower in NRCC-shFSTL1 cells than in NRCC-shScramble cells (55.5% ± 6.8% vs. 85.0% ± 1.2%). shFSTL1, *FSTL1* knockdown; shScramle, vector control. Each flow cytometry assay was conducted in triplicate and a representative figure is shown

In cell cycle assay, NRCC-shFSTL1 and NRCC-shScramble cells were cultured and passaged synchronously. Flow cytometry was applied to determine cell cycle and cellular markers. It was found that the percentage of cells gated in the S and G_2_/M phases were higher in NRCC-shFSTL1 cells than in NRCC-shScramble cells (*P* = 0.048, *P* = 0.011; Fig. 3a); the percentage of CD99-positive cells was increased (*P* = 0.026; Fig. 3b), whereas the percentage of CD24-positive cells was decreased in NRCC-shFSTL1 cells (*P* = 0.013; Fig. 3c). NRCC cells were nearly 100% positive for CD44 but almost all negative for CD133, CD105, EpCAM, and vimentin (data not shown), as measured by flow cytometry.

### *FSTL1* knockdown up-regulated the NF-κB and HIF signaling pathways

**Fig. 4:**
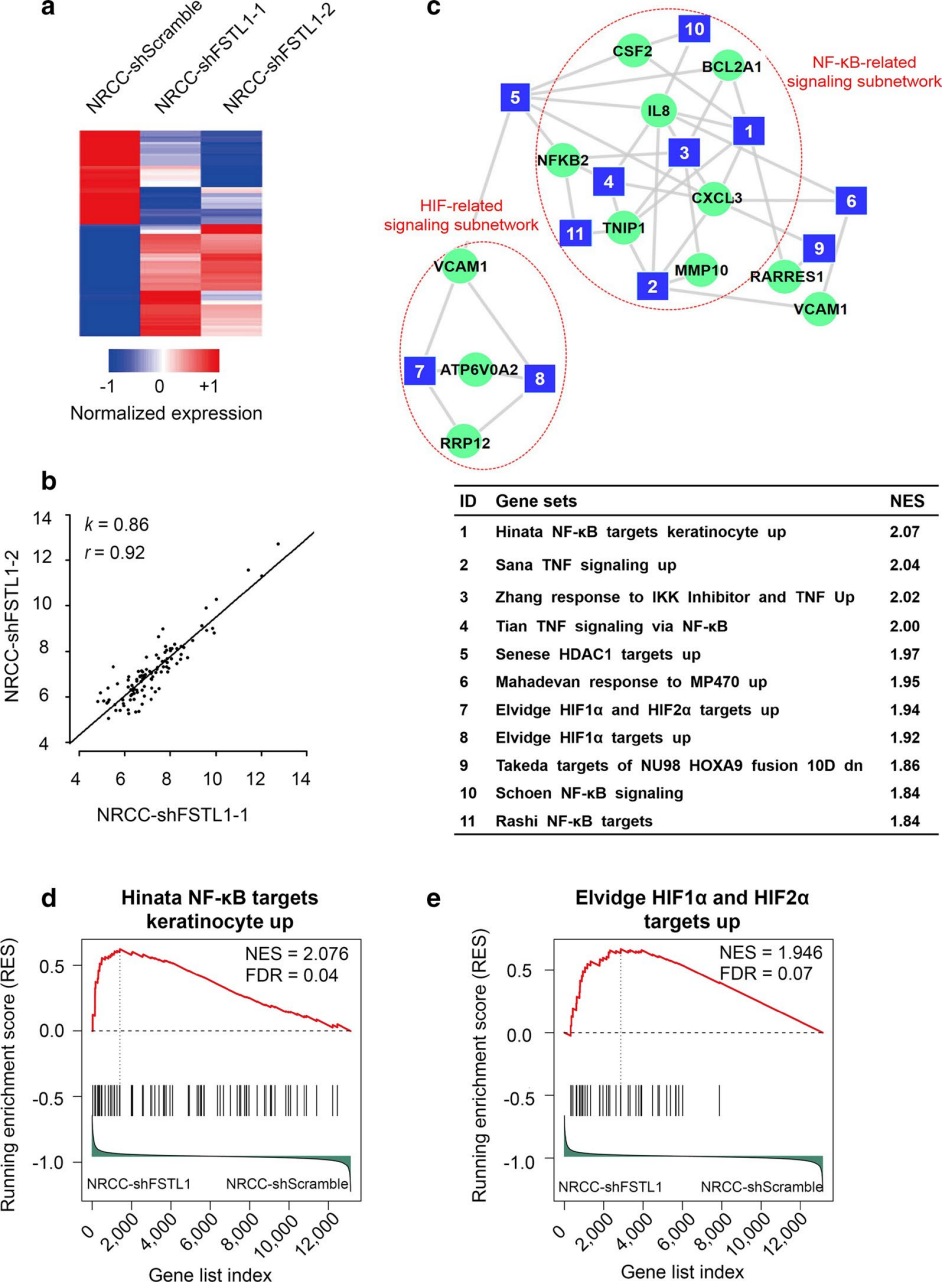
Major signaling pathways enriched by up-regulated genes in *FSTL1*-knockdown ccRCC cells by microarray assay. **a** Heat map of 105 differentially expressed genes (48 down-regulated and 57 up-regulated). **b** Reproducibility of the 105 differentially expressed genes in NRCC-shFSTL1-1 and NRCC-shFSTL1-2 cells (Pearson correlation coefficient *r* = 0.92; scope of linear model *k* = 0.86, *P* < 0.001). **c** Ranked by normalized enrichment score (NES), the top 11 gene sets (blue squares) enriched by up-regulated genes in response to *FSTL1* knockdown are selected to plot the network. Intersection genes (green dots) with fold change > 2 were plotted to connect the gene sets. The gene sets fell into nuclear factor-кB (NF-κB)- and hypoxia-inducible factor (HIF)-related signaling subnetworks, respectively, which is connected by a histone deacetylase 1 (HDAC1)-related gene sets. **d** Enrichment plot of the representative gene set with the highest NES score in the NF-κB-related signaling subnetwork. **e** Enrichment plot of the representative gene set with the highest NES score in the HIF-related signaling subnetwork. The vertical dashed line in each plot denoted the point at which NES reached its maximum

To explore the molecular mechanism by which FSTL1 affects the progression of ccRCC, we examined the gene expression profiles of the two NRCC-shFSTL1 cell lines with NRCC-shScramble cells using cDNA microarray assays (GEO Accession No. GSE76948). After filtered with absent/present calls, 105 differentially expressed genes with a fold change of > 2 were identified in *FSTL1* knockdown NRCC cells, including 57 up-regulated genes and 48 down-regulated genes. The spectrum and absolute gene expression levels of differentially expressed genes identified were consistent between NRCC-shFSTL1-1 and NRCC-shFSTL1-2 cells (Fig. 4a, b). We applied GSEA software package to enrich the gene sets from the global gene expression in response to *FSTL1* knockdown in NRCC cells. Taking FDR of < 25% as threshold, 12 functional gene sets were enriched by the down-regulated genes and 23 were enriched by the up-regulated genes. According to NES, the top 11 gene sets enriched by the up-regulated genes were applied to construct a network (Fig. 4c). In this network, NF-κB- and HIF-related functional gene sets formed two obvious subnetworks. The representative gene sets with the highest NES score in the NF-κB- and the HIF-related signaling subnetworks were named “Hinata NF-κB targets keratinocyte up” [35] and “Elvidge HIF1α and HIF2α targets up” [36], respectively (Fig. 4d, e).

### *FSTL1* knockdown promoted *IL*-*6* expression, epithelial-to-mesenchymal transition (EMT), and TNFα-induced degradation of NF-κB inhibitor (IκBα) in ccRCC cells

**Fig. 5:**
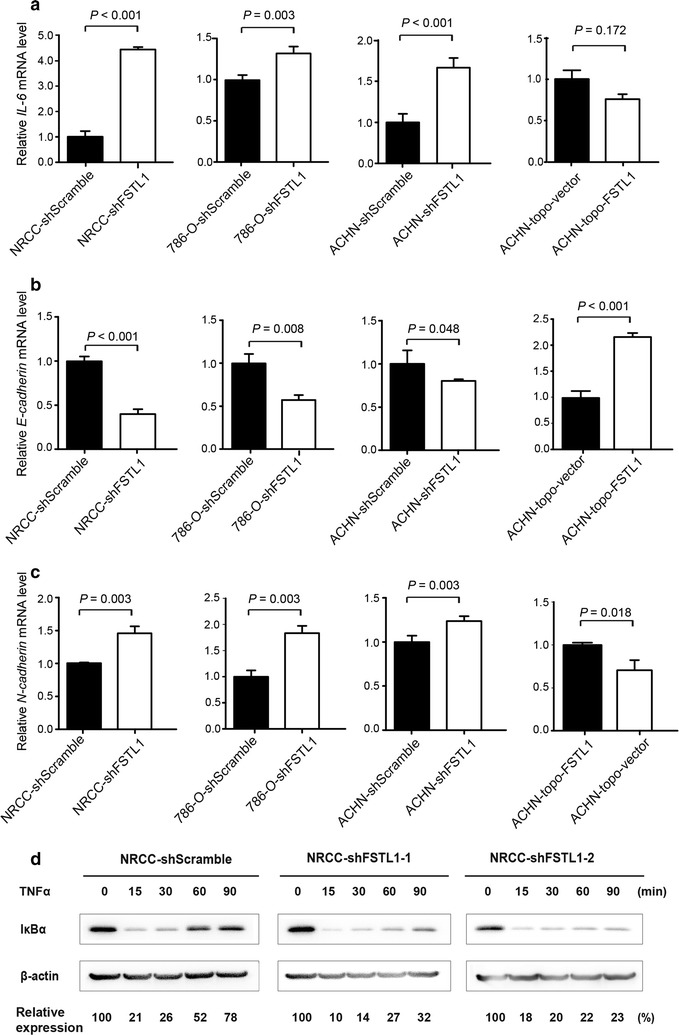
Effect of *FSTL1* knockdown on epithelial-to-mesenchymal transition (EMT) and NF-κB signaling. **a** Levels of *IL*-*6* mRNA in NRCC-shFSTL1, 786-O-shFSTL1, ACHN-shFSTL1, and in ACHN-topo-FSTL1. **b** Levels of *E*-*cadherin* mRNA in ccRCC cell lines with *FSTL1* knockdown and in ACHN cells with topo-FSTL1. **c** Levels of *N*-*cadherin* mRNA in ccRCC cell lines with *FSTL1* knockdown and in ACHN cells with topo-FSTL1. **d** *FSTL1* knockdown speeded up the degradation of NF-κB inhibitor (IκBα) in ccRCC cells at 60 min following TNFα treatment. The relative expression of IκBα at 60 min is higher in NRCC-shScramble cells (57.33 ± 4.37, *n* = 3) than in NRCC-shFSTL1-1 cells (36.67 ± 5.84, *n* = 3, *P* = 0.036) and NRCC-shFSTL1-2 cells (33.67 ± 5.04, *n* = 3, *P* = 0.032)

Our qRT-PCR assays indicated that *IL*-*6* transcription was significantly up-regulated following *FSTL1* knockdown in ACHN, 786-O, and NRCC cells (*P* < 0.05) (Fig. 5a). The transcription of *E*-*cadherin* was significantly down-regulated whereas *N*-*cadherin* was significantly up-regulated following *FSTL1* knockdown in NRCC cells; transient transfection of shFSTL1 also down-regulated *E*-*cadherin* and up-regulated *N*-*cadherin* in ACHN cells. Furthermore, transient transfection of *FSTL1* cDNA significantly up-regulated *E*-*cadherin* and down-regulated *N*-*cadherin* in ACHN cells (Fig. 5b, c). We used Western blotting to analyze the expression of IκBα protein in response to TNFα (10 ng/mL) treatment for 0, 15, 30, 60, and 90 min. The relative IκBα levels were calculated based on its baseline expression at the 0 min and adjusted by expression levels of β-actin. It was found that *FSTL1* knockdown repressed the recovery of IκBα after TNFα treatment (Fig. 5d).

### Expression patterns of FSTL1, HIF-1α, and HIF-2α in human ccRCC tissues and adjacent renal tissues as well as their roles in predicting postoperative prognosis

**Fig. 6:**
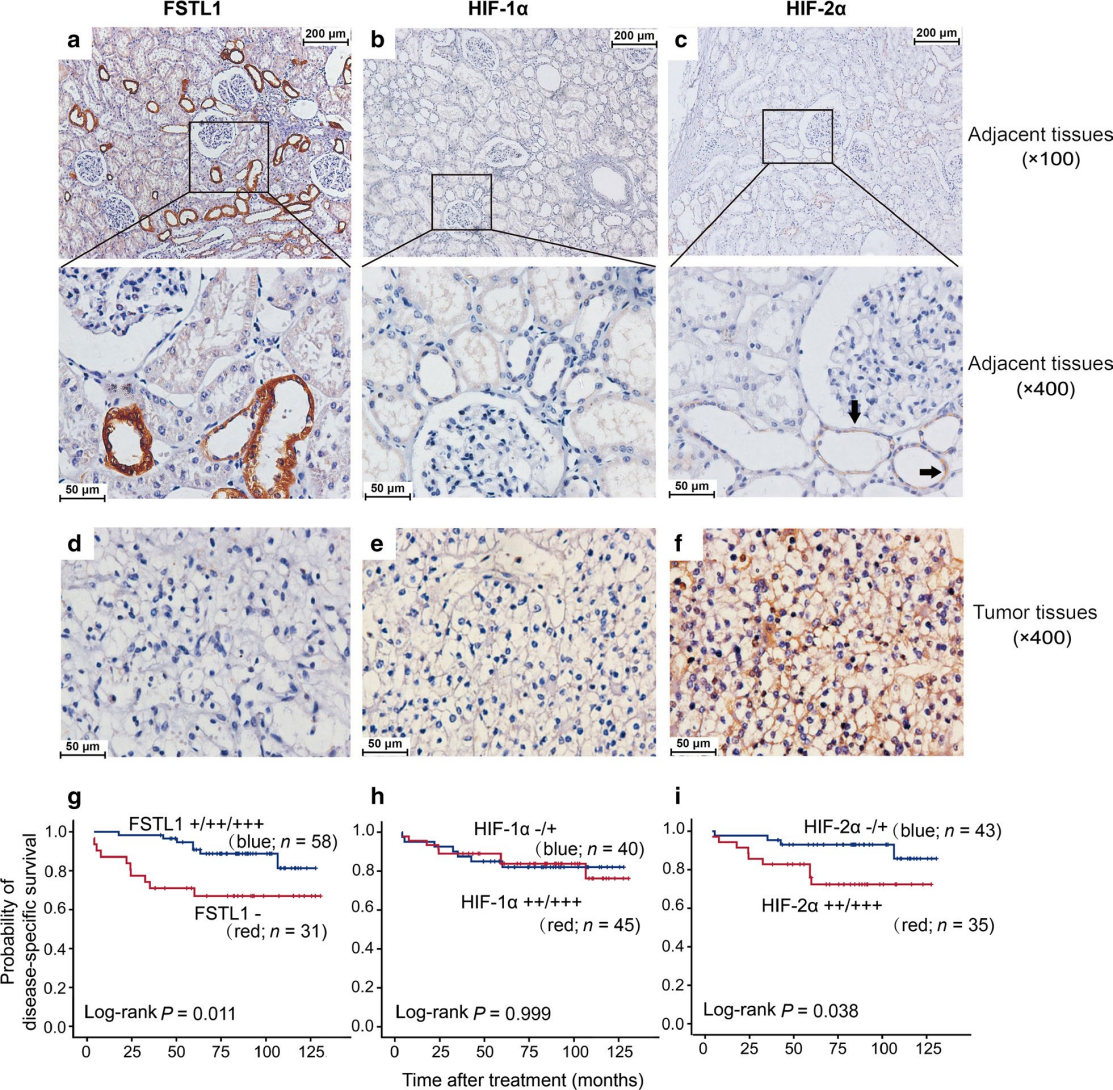
FSTL1 and HIF-1α/2α levels in ccRCCs and adjacent tissues and their effects on postoperative prognosis. **a**–**c** Representative immunostaining of FSTL1, HIF-1α, and HIF-2α in adjacent renal tissues, respectively. The HIF-2α positive parts are indicated by black arrows. **d**–**f** Representative immunostaining of FSTL1, HIF-1α, and HIF-2α in ccRCC tissues, respectively. **g**–**i** Effect of intratumoral expression of FSTL1, HIF-1α, and HIF-2α on disease-specific survival, respectively

A total of 89 ccRCC patients (men, 59; women, 30) were involved in the final analysis. The median age of the 89 ccRCC patients was 59 years (range 25–85 years). Sixty-three (70.8%) patients had stage I-II ccRCC. The median follow-up time was 82.3 months (interquartile range 58.8–102.2 months). All patients involved in this study had tumor tissue samples and 67 had the paired adjacent renal tissues. FSTL1 expression was examined by using IHC in all 89 tumor tissues, and 67 adjacent renal tissues. However, HIF-1α and HIF-2α expression was examined in only 85 and 78 tumor tissues, respectively, because some tissue specimens were insufficient for IHC assays of all target proteins. FSTL1 was positive in 65.2% (58/89) of ccRCC tissues and 94.0% (63/67) of the adjacent non-cancer tissues. In the adjacent tissues, FSTL1 was selectively positive in epithelial cytoplasm of the loop of Henle; HIF-1α was negative whereas HIF-2α was positive in the loop of Henle (Fig. 6a–c). In ccRCC tissues, FSTL1 was negative or detected in the cytoplasm of cancer cells; HIF-1α and HIF-2α were positive in 52.9% (45/85) and 66.7% (52/78) of ccRCC cases, respectively (Fig. 6d–f). The positive rate of FSTL1 protein was significantly lower in ccRCC tissues than in the adjacent normal tissues among 67 patients with paired ccRCC specimens (*P* < 0.001, Table 2). In ccRCC tissues, FSTL1 expression was positively correlated with HIF-1α (Spearmen *r* = 0.216, *P* = 0.047) but negatively correlated with HIF-2α expression (Spearmen *r* = − 0.229, *P* = 0.044) (Table 3).

**Table 2:** Comparison of FSTL1 expression between tumor and paired adjacent tissues of 67 patients with clear-cell renal cell carcinoma

Tissue type	Immunoreactive scores of FSTL1 [cases (%)]				Mean rank	*P*
	−	+	++	+++		
Cancer tissues	28 (41.8)	29 (43.3)	4 (6.0)	6 (9.0)	49.50	< 0.001
Paired normal tissues	4 (6.0)	25 (37.3)	20 (29.9)	18 (26.9)	85.50	–

*FSTL1* follistatin-like protein 1

**Table 3:** The correlation of FSTL1 level with the expression of HIF-1α/2α in ccRCC tissues and tumor stage

Variable	Immunoreactive scores of FSTL1 (cases)		*P*	*r*_*s*_
	Negative	Positive		
HIF-1α expression				
Negative (−)	19	21	0.047	0.216
Positive (+/++/+++)	12	33		
HIF-2α expression				
Negative (–)	5	21	0.044	− 0.229
Positive (+/++/+++)	22	30		
AJCC stage				
I	16	39	0.110	− 0.170
II	3	5		
III	6	9		
IV	6	5		

*ccRCC* clear-cell renal cell carcinoma; *FSTL1* follistatin-like protein 1; *HIF* hypoxia-inducible factor; *AJCC* American Joint Committee on Cancer; *r*_*s*_ Spearman rank correlation coefficient

A total of 89 ccRCC patients who had IHC scores of FSTL1 expression in tumor tissues were successfully followed up after surgery. Up to the date of last visit, 17 patients died of ccRCC relapse. Kaplan–Meier analysis indicated that intratumoral FSTL1 expression predicted a favorable postoperative DSS (Fig. 6g). The Kaplan–Meier survival curves for FSTL1-positive and FSTL1-negative patients were not cross, suggesting that the proportional hazards assumption was reasonable in this case. Compared with the patients with AJCC stages III–IV ccRCC, those with stages I–II diseases had a good prognosis (log-rank test, *P* = 0.004). No significant correlation was observed between intratumoral FSTL1 expression and AJCC stage of ccRCC patients (Table 3). Multivariate Cox regression analysis including age, gender, and AJCC stage showed that intratumoral FSTL1 expression conferred a favorable postoperative prognosis independently (Table 4). Moreover, Kaplan–Meier analysis showed that high expression of HIF-2α, rather than HIF-1α, in tumor tissues predicted an unfavorable postoperative prognosis (Fig. 6h, i).

**Table 4:** Factors predicting disease-specific survival of ccRCC patients in multivariate Cox proportional hazards model

Variable	HR (95% CI)	*P*
Age (≤ 59 vs. > 59 years)	0.992 (0.953–1.033)	0.696
Gender (male vs. female)	1.337 (0.470–3.802)	0.585
AJCC stage (III–IV vs. I–II)	3.704 (1.376–9.974)	0.010
FSTL1 (positive vs. negative)	0.325 (0.118–0.894)	0.030

*AJCC* American Joint Committee on Cancer; *FSTL1* follistatin-like protein 1; *HR* hazard ratio; *ccRCC* clear-cell renal cell carcinoma

## Discussion

In the present study, we presented a series of data to identify that FSTL1 functioned as a novel tumor suppressor in ccRCC. However, related evidence was mostly obtained from *FSTL1* knockdown assays. *FSTL1* knockdown facilitated ccRCC cell growth, migration, invasion, and promoted cell cycle, possibly because a low level of FSTL1 was unable to repress some cancer-promoting force(s); overexpression of *FSTL1* did not inhibit the growth and invasion, possibly because FSTL1 expression at the normal level was enough to repress the cancer-promoting force(s), thus it should be important to restore the FSTL1 expression level to the normal range for attenuating the growth advantage of ccRCC. We found that MRCC and ACHN, the two cell lines derived from metastatic ccRCC tissues, were resistant to the infection with recombinant retrovirus; furthermore, MRCC was also resistant to the transient transfection, which is possibly due to the “stem-like” property of metastatic ccRCC cells [30]. Thus, we gave up our intention to up-regulate the expression of FSTL1 in MRCC. Our cytometry results indicated that *FSTL1* knockdown up-regulated CD99, a cellular marker related to ccRCC aggressiveness [37], and down-regulated CD24 expression in NRCC cells. We found that CD44 was nearly 100% positive in NRCC. High CD44 expression in tumors is a poor prognostic marker of RCC [12]. CD44^high^CD24^low^ signature usually determines the cancer stem cell (CSC) and EMT phenotype in oral cancer [38]. Additionally, CD44^high^CD24^low^ signature is mostly believed to be a CSC-like phenotype in cancers such as prostate cancer [39] and breast cancer [40]. Thus, it is possible that *FSTL1* knockdown increases CSC-like properties of ccRCC cells. We also found that *FSTL1* knockdown promoted EMT process via up-regulating *N*-*cadherin* and down-regulating *E*-*cadherin* in ccRCC cells. Thus, FSTL1 might be a novel tumor suppressor that attenuates EMT process in ccRCC cells.

Using global gene expression profiling, we found NF-κB- and HIF-related functional gene sets were the predominant signaling pathways affected by *FSTL1* knockdown. Interestingly, a histone deacetylase 1 (HDAC1)-related pathway bridges the NF-κB-related signaling subnetwork and HIF-related signaling subnetwork. This is supported by previous findings [41]. HDAC1 may link the NF-κB and HIF signaling pathways. The treatment with HDAC1 inhibitors might be an option of advanced RCC [42]. The two pathways work synergistically in tumor cells that survive under both inflammatory and hypoxia microenvironments. Thus, we chose to validate the activity of the NF-κB signaling pathway in ccRCC cells and the expression of HIF1/2α in clinical samples. *FSTL1* knockdown up-regulated the expression of *IL*-*6* in ccRCC cells. Elevated level of *IL*-*6* expression is associated with cancer cell proliferation, angiogenesis, and metastasis via fueling signal transducer and activator of transcription 3 (STAT3), mitogen-activated protein kinase (MAPK), and Akt signaling, thus promoting EMT and subsequent cancer metastasis [43]. *FSTL1* knockdown promotes the NF-κB signaling pathway in ccRCC cells (Fig. 5d), whereas activation of NF-κB promotes the development of RCC [44]. *FSTL1* knockdown also affect the HIF signaling pathway. HIF-1α and HIF-2α have opposing effects in ccRCC biology, with HIF-1α acting as a tumor suppressor and HIF-2α acting as an oncogene [45]. The somatic mutation of von Hippel–Lindau (*VHL*) gene, a tumor suppressor gene, can lead to increased expression of HIF-2α in both sporadic and familial ccRCCs [46]. HIF-2α activation is involved in the generation of RCC-derived, CXCR4-positive CSCs [47]. Therefore, we believe that *FSTL1* knockdown may derepress NF-κB and HIF-2α signaling in ccRCC cells, thus promoting cancer invasion and metastasis. Interestingly, both FSTL1 and HIF-2α were locally expressed at the same locations but FSTL1 expression was negatively associated with HIF-2α expression in ccRCC tissues, implying that FSTL1 in situ inhibit HIF-2α expression in renal tissues. The mechanisms by which FSTL1 in situ inhibit HIF-2α expression remains to be elucidated.

Our IHC analyses indicated that FSTL1 was locally expressed in epithelial cytoplasm in the loop of Henle, which is consistent with the results of a previous study [32]. However, it is mainly expressed in epithelial cells, rather than in mesenchymal cells as previously believed [22], indicating that the role of FSTL1 in renal tissues is quite special. FSTL1 expression is significantly lower in ccRCC than in adjacent renal tissues, but the intratumoral FSTL1 expression is not associated with tumor stage. Importantly, intratumoral FSTL1 expression confers a favorable independent postoperative prognosis in ccRCC; whereas high expression of HIF-2α, rather than HIF-1α, in ccRCC tissues predicts a poor postoperative prognosis. Thus, intratumoral FSTL1 and HIF-2α expression are both prognostic factors for ccRCC; however, they have inverse effects.

Our study has limitations. First, the cancer inhibition effect of FSTL1 was investigated only in vitro. The evidence generated from animal experiments are needed to further clarify the function of FSTL1 on the growth and metastatic potential of ccRCC cells. Second, only 89 patients were enrolled in this study. The value of FSTL1 for prognosis prediction remains to be re-confirmed in large cohorts.

## Conclusions

This present study clarifies the important nature of FSTL1 in ccRCC. FSTL1 functions as a tumor suppressor possibly via repressing NF-κB and HIF-2α signaling pathways. Intratumoral FSTL1 expression in ccRCC tissues conferred a favorable independent postoperative prognosis to ccRCC patients. Treatment approaches that increasing FSTL1 expression in tumors might lead to effective therapy for advanced ccRCC.

## Abbreviations

FSTL1: follistatin-like protein 1
RCC: renal cell carcinoma
ccRCC: clear cell RCC
HIF: hypoxia-inducible factor
IκBα: inhibitor of NF-κB
CXCR8: chemokine (C-X-C motif) receptor 8
CXCL16: chemokine (C-X-C motif) ligand 16
IL-6: interleukin-6
TNFα: tumor necrosis factor-α
CRC: colorectal cancer
FBS: fetal bovine serum
GAPDH: glyceraldehyde-3-phosphate dehydrogenase
IHC: immunohistochemistry
HRs: hazard ratios
CIs: confidence intervals
HEK 293T: human embryonic kidney 293T cells
shRNA: short hairpin RNA
GSEA: gene set enrichment analysis
FDRs: false discovery rates
NES: normalized enrichment scores
EMT: epithelial-to-mesenchymal transition
AJCC: American Joint Committee on Cancer
DSS: disease-specific survival
CSC: cancer stem cell
HDAC1: histone deacetylase 1
STAT3: signal transducer and activator of transcription 3
MAPK: mitogen-activated protein kinase
VHL: von Hippel–Lindau gene
